# The Prince of Wales at St. Bartholomew's Hospital

**Published:** 1901-12-14

**Authors:** 


### THE PRINCE OF WALES AT ST. BARTHOLOMEW'S HOSPITAL.

All who take an interest in hospital work and who recognise how largely that work has been assisted during the past quarter of a century by the influence of the Royal Family, and more especially by the personal participation of our King in matters of hospital management, will be glad to find that the Prince of Wales is taking up the work with which his royal father has been so long engaged.

Last week the Prince of Wales visited St. Bartholomew's Hospital and attended a special court of the governors for the purpose of receiving his charge as president of the hospital. His Royal Highness, who was attended by the Hon. Derek Keppel as equerry in waiting, was greeted on arrival at the hospital with loud cheers by the students who were assembled in the quadrangle. He was received by Sir Trevor Lawrence (the treasurer), the four almoners—Mr. Grey, Mr. Cooper, Mr. Alliston, and Mr. Baker—and the clerk, Mr. W. H. Cross. Conducted by these gentlemen, the Prince of Wales proceeded to the great hall, where a numerous company had assembled, including the Lord Mayor, Mr. Alderman and Sheriff Bell, Alderman Sir J. Whittaker Ellis, Alderman Sir A. Hanson, Alderman Sir James Ritchie, Alderman Vaughan Morgan, Alderman Smallman, Alderman Crosby, the Rev. Sir Borradaile Savory, Sir James Blyth, Mr. B. L. Cohen, M.P., Sir E. Durning-Lawrence, M.P., Sir Dyce Duckworth, Sir William Church (senior physician), Mr. Alfred Willettt (senior surgeon), Mr. John Langton, Mr. Onslow Ford, R.A., and practically the whole of the medical and surgical staff, with the matron and the sisters of the wards, Mr. E. B. I'Anson (surveyor), Mr.

E. J. Wilde (solicitor), and Mr. H. Wingfield Cross (assistant secretary).

In the great hall the chair was taken by the treasurer, the Prince of Wales being seated on his right hand, and the Lord Mayor on his left.

Sir Trevor Lawrence addressing his Royal Highness, gave a short history of the hospital which he believed he might say was the oldest institution of the sort within the limits of the Empire. After bearing testimony to the great interest his Majesty the King had ever taken, during his long presidency of 34 years, in that hospital, and to the valuable services he had rendered it on many occasions, he said that his Majesty was so good as to preside over a court of that hospital in July of last year and to give his valuable help towards securing an addition to the site of that hospital. In conclusion, on behalf of the governors and the staff of all degrees, of the citizens of London, and of the poor patients who were harboured and treated in that hospital, he begged to tender to his Royal Highness their most grateful thanks for the gracious and ready kindness with which he assented to the proposal that he should become their president. They hoped his Royal Highness would long rule over them as their president, and that he would show in that institution the same interest which had been so long and constantly shown by his Majesty the King. (Loud cheers.)

The Secretary (Mr. W. H. Cross) then read the governor's charge to his Royal Highness. The following was the charge as a governor:—

Your Royal Highness having been elected and chosen a governor of St. Bartholomew's Hospital, it is your duty and charge to acquit yourself in that office with all faithfulness and sincerity, endeavouring that the affairs and business of the said hospital may be well ordered and managed and promoting the weal and advantage of the poor, wounded, sick, maimed, diseased persons harboured in the said hospital. To this end your Royal Highness is now admitted a governor of St. Bartholomew's Hospital.

Mr. Almoner Grey then handed to his Royal Highness a copy of the governor's charge, Mr. Almoner Cooper handed to him the governor's staff, Mr. Almoner Alliston the "rules and orders" of the hospital, and Mr. Almoner Baker the calendar of the hospital's medical school.

The Treasurer then asked his Royal Highness whether he would be pleased to receive his charge as president.

The Prince of Wales having intimated his assent, the clerk read the charge as follows:—

Sir,—Having been elected president of this hospital, your Royal Highness has to be received as its chief ruler and governor. Your Royal Highness is to convene general courts at such times as you may deem necessary or be required by a resolution of the house committee, or by a requisition in writing signed by 13 governors at the least; and your Royal Highness is to preside at the same, as well as at committees and on all other public occasions when you may think proper to attend.

A copy of the president's charge was handed to the Prince of Wales by the treasurer, who then vacated the chair in his Royal Highness's favour.

The Prince of Wales, who was received with cheers, having taken the chair, said:—

Sir Trevor Lawrence, my Lord Mayor, and Gentlemen,—I thank you for the great honour you have done me in electing me president of your hospital. I can only assure you that I am proud to become associated with an institution whose history dates back to the twelfth century, and which, ever since then, has worked for the relief of the pain and suffering amongst the poor of the City of London. I look upon it as a very great compliment to have been asked to succeed my dear father—(cheers)—who, as Sir Trevor has just told us, held the position of your president for 34 years. (Cheers.) He certainly took, during that time, the keenest interest in the welfare of St. Bartholomew's Hospital, and he wishes me to assure you that that interest will be maintained. (Cheers.) I fully realise the responsibility of the trust which you have to-day confided in me, and you may certainly count upon my services being always readily given to further the good work of this institution, with which I so heartily sympathise (Loud cheers.)

His Royal Highness inquired whether there were any governors present who had not yet received their charge, and Mr. Hill, a new governor, came forward to receive his charge, which was read to him by the clerk, a copy of it being handed to him by the president.

The formal proceedings were now at an end, but before leaving the great hall the Prince of Wales inspected a marble bust of Queen Victoria which had been executed by Mr. Onslow Ford, R.A., and presented to the hospital by Mr. Ebenezer Homan, one of the governors. His Royal Highness shook hands with several of the governors and with Mr. Onslow Ford and other gentlemen, and then left the building. As he drove away he was again heartily cheered by the large number of students who had assembled.

